# Influencing Factors of Hospital-Acquired Pneumonia Infection in the Middle-Aged and Elderly Patients With Schizophrenia

**DOI:** 10.3389/fpsyt.2021.746791

**Published:** 2021-10-15

**Authors:** Mi Yang, Qiwen Li, Chunzhi Wang, Li Li, Min Xu, Fei Yan, Wei Chen, Ying Wan

**Affiliations:** ^1^The Fourth People's Hospital of Chengdu, Chengdu, China; ^2^Ministry of Education (MOE) Key Lab for Neuroinformation, The Clinical Hospital of Chengdu Brain Science Institute, University of Electronic Science and Technology of China, Chengdu, China; ^3^Ministry of Education (MOE) Key Laboratory for Neuroinformation, High-Field Magnetic Resonance Brain Imaging Key Laboratory of Sichuan Province, University of Electronic Science and Technology of China, Chengdu, China; ^4^Qingdao Mental Health Center, Qingdao University, Qingdao, China

**Keywords:** elder, schizophrenia, hospital-acquired pneumonia, antipsychotics, clozapine

## Abstract

**Introduction:** Pneumonia is an important cause of death in patients with schizophrenia. It is critical to understand the risk factors of hospital-acquired pneumonia (HAP) and determine prevention strategies to reduce HAP. The aim of this study is to elucidate the risk factors for HAP in the middle-aged and elderly hospitalized patients with schizophrenia.

**Methods:** We retrospectively reviewed the medical records of 2,617 the middle-aged and elderly patients (age ≥ 50) with schizophrenia who were admitted for the first time to a large-scale psychiatric hospital between 2016 and 2020. The factors related to the incidence of HAP in patients were analyzed, including personal characteristics, antipsychotics, and non-antipsychotics.

**Results:** The HAP infection rate of hospitalized the middle-aged and elderly patients with schizophrenia was 7.8%. Chi-square analyses showed that older age, male, and ≥60 days of hospitalization were risk factors for HAP infection (χ^2^ = 94.272, *p* < 0.001; χ^2^ = 22.110, *p* < 0.001; χ^2^ = 8.402, *p* = 0.004). Multivariate logistic regression showed that quetiapine, clozapine, and olanzapine significantly increased the incidence of HAP (OR = 1.56, 95% CI = 1.05–2.32, *p* = 0.029; OR = 1.81, 95% CI = 1.26–2.60, *p* = 0.001; OR = 1.68, 95% CI = 1.16–2.42, *p* = 0.006). Antipsychotic drugs combined with aceglutamide had an effect on HAP (OR = 2.19, 95% CI = 1.38–3.47, *p* = 0.001).

**Conclusion:** The high HAP infection rate in hospitalized the middle-aged and elderly patients with schizophrenia may be related to the increase of age and the use of antipsychotic drugs. The types and dosages of antipsychotic drugs should be minimized while paying attention to the mental symptoms of patients.

## Introduction

Hospital-acquired infections (HAI) are infections acquired at least 48 h or beyond the average incubation period after admission, not present or incubating at the time of admission (1, 2). Hospital-acquired pneumonia (HAP) is one of the most common nosocomial infections (3, 4), accounting for about 20–40% (5–7) of HAI. Previous evidence has suggested that aspiration pneumonia due to aspiration is an important mechanism for the pathogenesis of pneumonia in elder people (8–10). When aspiration occurs, oropharyngeal or gastric material is usually misdirected into the lower respiratory tract due to dysphagia or ineffective cough (11).

Studies (12, 13) have shown that schizophrenic patients, due to mental consciousness disorder and cognitive status abnormality, has higher risks of other body diseases and more difficulty obtaining better treatment effect (14) than the general population. Elderly patients with severe schizophrenia who need hospitalization are more likely to suffer from HAI, especially respiratory tract infection, due to long-term bedridden, mandatory restraint, decreased immunity, and other factors (15–17). In particular, the incidence of HAP in elderly patients with schizophrenia is higher than that in the general population (OR = 1.7) (18), and HAP is one of the main causes of death in hospitalized patients with schizophrenia (19, 20).

Leischker et al. proposed that the reduced immune function of people aged over 50 is associated with an increased risk of infectious diseases (21). Foppa et al. (22) found that the death rate from influenza increases with age over 50 and is highest in people aged over 65. Merzon's case-control study of 7,807 patients with COVID-19 found that the incidence of COVID-19 is associated with age over 50 years (23). Other studies have reported that the incidence of invasive pneumococcal disease is highest in people under 5 and over 50 years of age (21). Further researches have shown that being older than 50 is a risk factor for death from pneumonia (22, 24).

Therefore, this study analyzes the relationship between multiple indicators of the middle-aged and elderly schizophrenia patients aged 50 or above and HAP in a large-scale psychiatric hospital, in order to assess the risk factors of HAP infection in inpatients and thus formulate corresponding prevention strategies since prevention strategies based on modifiable risk factors are important for reducing HAP-related mortality in elderly patients with schizophrenia (25).

## Methods and materials

### Sample Selection and Population

The clinical data of consecutive patients admitted to a large-scale psychiatric hospital during a 4-year period from January 2016 to December 2020 were retrospectively reviewed. Patients with schizophrenia hospitalized for 3 days or more during a 5-year period were included. The diagnostic criteria were consistent with the primary diagnosis of schizophrenia (ICD-10). Other inclusion criteria included first admission, age (≥50 years), and use of any antipsychotic medication during hospitalization. The exclusion criterion was that the patient's primary information was incomplete. Subsequently, 2,617 patients with schizophrenia were enrolled. Medications (antipsychotics and non-antipsychotics), epidemic data including age, sex, marriage, hospital stay, diabetes, hypertension, and whether they had HAP were collected. This study was approved by the Institutional Review Committee of the Fourth People's Hospital of Chengdu and was designed retrospectively without written informed consent.

### Use of Medicines

The second-generation antipsychotics (SGA) used in this study included clozapine, olanzapine, quetiapine, risperidone, amisulpride, ziprasidone, aripiprazole, and paliperidone. First-generation antipsychotics (FGA) included chlorpromazine, perphenazine, haloperidol, flupentixol, and sulpiride.

The non-antipsychotic drugs used in this study included sedative-hypnotic, antianxiety, antimanic, antidepression, antiepileptic, antiparkinsonian, and other neuron drugs (aceglutamide).

### Statistical Analysis

Individual differences between patients with and without HAP were analyzed using Chi-square tests for categorical variables. The *Bonferroni* method was used to adjust the level of α for pairwise comparison. Univariate conditional logistic regression was initially used to compare drug use between patients with and without HAP. Covariates reasonably (*p* < 0.05) associated with HAP infection were then input into the final adjustment model. Multivariate conditional logistic regression was used to adjust the model and assess the impact of individual antipsychotics on the risk of HAP infection. SPSS 24 was used for the analyses. *p* < 0.05 was considered significant.

## Results

### Personal Characteristics of Schizophrenia Patients

The mean age of the 2,617 the middle-aged and elderly patients with schizophrenia was 59.35 ± 7.85 years. In the patients, 203 cases were infected with HAP. The infection rate was 7.8%. Males accounted for 35.1% and females accounted for 64.9% of the HAP infected patients. The rate of infection significantly increased with age (χ^2^ = 94.272, *p* < 0.001) and was higher in males than that in females (χ^2^ = 22.110, *p* < 0.001). The rate differed between those hospitalized for less and more than 60 days (χ^2^ = 8.402, *p* = 0.004; Table 1).

**Table 1:** Personal characteristics of HAP infection or not in hospitalized patients with schizophrenia (*N* = 2,617).

**Variable**	**HAP Infections**					**χ^2^**	***p*-value**
		**No (N)**	**Percentage (%)**	**Yes (N)**	**Percentage (%)**		
Age	50~59	1,411	95.7	63	4.3	94.272	0.000
	60~69	758	90.3	81	9.7		
	70~79	214	82.3	46	17.7		
	≥80	31	70.5	13	29.5		
Gender	Male	817	88.9	102	11.1	22.110	0.000
	Female	1,597	94.1	101	5.9		
Marital status	Married	1,360	92.8	105	7.2	1.627	0.443
	Single	275	91.4	26	8.6		
	Others	779	91.5	72	8.5		
Diabetes	No	2,067	92.6	164	7.4	3.485	0.062
	Yes	1,120	93.6	77	6.4		
Hypertension	No	1,998	92.5	161	7.5	1.550	0.213
	Yes	416	90.8	42	9.2		
Hospital stay^*^	3~6^a^	102	94.4	6	5.6	8.716	0.069
	7~13^a^	256	92.8	20	7.2		
	14~29^a^	746	93.1	55	6.9		
	30~59^a^	691	93.1	51	6.9		
	≥60^b^	619	89.7	71	10.3		
The number of medications	1	844	92.2	71	7.8	0.294	0.863
	2	1,129	92.5	92	7.5		
	3 or more	441	91.7	40	8.3		
Total		2,414	92.2	203	7.8		

a,b *Indicate that the difference between groups (a and b) was statistically significant after Bonferroni correction*.

* *Indicate significant differences when patients with hospitalization days <60 days were combined into one group and compared with ≥60 days (χ^2^ = 8.402, p = 0.004)*.

### Effect of Antipsychotics on HAP Infection

There were significant differences in HAP infection among patients taking different antipsychotic drugs. Patients taking SGA had a significantly higher incidence of HAP (Table 2). The multivariate logistic regression analysis showed that SGA, notably quetiapine, clozapine, and olanzapine, significantly increased the incidence of HAP, and the risk was 1.5–1.8 times higher than it was when these drugs were not used (95% CI = 1.05–2.32, *p* = 0.029; 95% CI = 1.26–2.60, *p* = 0.001; 95% CI = 1.16–2.42, *p* = 0.006).

**Table 2:** Risk analysis of antipsychotic drug use and HAP infection.

**Characteristic *N* (%)**	**Crude OR**	**95% CI**	***p-*value**	**Adjusted OR**	**95% CI**	***p-*value**
**FGA**						
Chlorpromazine	3.28	0.91–11.84	0.070	2.24	0.59–8.53	0.236
Haloperidol	0.54	0.39–0.75	0.000	0.69	0.49–0.97	0.035
Flupentixol^a^	0.00	0.00	0.999	–	–	–
Perphenazine	1.12	0.34–3.68	0.856	0.94	0.27–3.25	0.926
Sulpiride	0.98	0.60–1.60	0.922	1.02	0.60–1.73	0.936
**SGA**						
Quetiapine	1.59	1.10–2.29	0.014	1.56	1.05–2.32	0.029
Clozapine	1.28	0.93–1.76	0.133	1.81	1.26–2.60	0.001
Olanzapine	1.65	1.19–2.29	0.003	1.68	1.16–2.42	0.006
Risperidone	1.11	0.82–1.50	0.512	1.39	0.98–1.99	0.066
Aripiprazole	0.58	0.29–1.14	0.114	0.65	0.32–1.33	0.237
Ziprasidone	0.31	0.08–1.26	0.100	0.69	0.16–2.91	0.611
Paliperidone	0.75	0.27–2.08	0.581	0.88	0.31–2.53	0.814
Amisulpride	0.68	0.25–1.89	0.463	0.92	0.32–2.65	0.882

*Crude OR (Crude odds ratio): Univariate conditional logistic regression results*.

*Adjusted OR (Adjusted odds ratio): Multivariate conditional logistic regression results, regression factors include: age, gender, FGA (chlorpromazine, haloperidol, perphenazine, and sulpiride), SGA (quetiapine, clozapine, olanzapine, risperidone, aripiprazole, ziprasidone, paliperidone, and amisulpride) and non-antipsychotic drugs (sedative-hypnotic, antidepression, and aceglutamide)*.

*CI, confidence interval*.

a *The infected number of people using the flupentixol was 0*.

### Effects of Non-antipsychotics on HAP Infection

Since all patients were on antipsychotics, the analysis of the effect of non-antipsychotics on the occurrence of HAP was in fact an analysis of the effect of the combination of non-antipsychotics and antipsychotics. The results showed that antipsychotic drugs combined with aceglutamide had an effect on HAP (OR = 2.19, 95% CI = 1.38–3.47, *p* = 0.001; Table 3).

**Table 3:** Risk analysis of non-antipsychotic drug use and HAP infection.

**Characteristic *N* (%)**	**Crude OR**	**95% CI**	***p-*value**	**Adjusted OR**	**95% CI**	***p-*value**
Sedative-hypnotic	0.69	0.52–0.93	0.013	0.89	0.64–1.23	0.474
Antianxiety	0.96	0.41–2.24	0.930	–	–	–
Antimanic^a^	0.00	0.00	0.998	–	–	–
Antidepression	1.58	1.06–2.37	0.026	1.53	0.99–2.37	0.057
Antiepileptic	1.39	0.92–2.09	0.115	–	–	–
Antiparkinsonian	0.85	0.62–1.16	0.293	–	–	–
Aceglutamide	2.35	1.53–3.61	0.000	2.19	1.38–3.47	0.001

*Crude OR (Crude odds ratio): Univariate conditional logistic regression results*.

*Adjusted OR (Adjusted odds ratio): Multivariate conditional logistic regression results, regression factors include: age, gender, FGA (chlorpromazine, haloperidol, perphenazine, and sulpiride), SGA (quetiapine, clozapine, olanzapine, risperidone, aripiprazole, ziprasidone, paliperidone, and amisulpride), and non-antipsychotic drugs (sedative-hypnotic, antidepression, and aceglutamide)*.

*CI, confidence interval*.

a *The infected number of people using the antimanic was 0*.

## Discussion

Augmented risks of HAP severely reduces the quality of life and increases the burden of disease and the risk of death in hospitalized psychiatric patients, especially those being over 50 years old (19). Numerous studies (26, 27) have focused on the risk factors for HAP in non-psychotic patients. In contrast, much fewer have address the issue in schizophrenia patients, none of which have concerned HAP risks in elder patients, who are particularly at high risk for HAI and in need of close attention.

### Mechanism and Infection Rate of HAP

Our study showed that the HAP incidence in hospitalized the middle-aged and elderly patients with schizophrenia was 7.8%, which is much higher than the overall HAP incidence of 2.33% in a Chinese meta-analysis (28). It is also higher than the HAP rate of 4.17% in schizophrenia patients in Liu et al. (29). Risk factors associated with HAP in this study were age, gender, hospital stay (≥60 days), SGA (quetiapine, clozapine, and olanzapine), and non-antipsychotic drugs (aceglutamide). These findings are basically consistent with the results in previous studies (18, 30, 31).

The ward of mental institutions usually adopts closed-off management, leading to limited space for activities of patients and poor indoor ventilation. Medical staffs specialize in different domains rather than respiratory diseases and patients themselves lack desire for treatment of the diseases, which increases the likelihood to delay the prevention and recognition of respiratory diseases and in turn increases HAP incidence. The patients enrolled in this study were the middle-aged and elderly patients with the age of over 50 years, whose tissues, organs, and immune system function showed a trend of gradual decline (32, 33), leading to a higher possibility of an increasing rate of HAI.

### Common Risk Factors for HAP

A significant positive correlation was found between age and HAP (χ^2^ = 94.272, *p* < 0.001). This may be due to the age-related changes in patients' body, e.g., the function of respiratory mucosal barrier and cough reflex (32, 33). Although the number of female schizophrenia patients was larger than that of males, the incidence of HAP in male patients was significantly higher than that in females (χ^2^ = 22.110, *p* < 0.001), which may be related to smoking and poor oral self-management in men. Patients with a hospital stay of ≥60 days had a higher HAP infection rate (χ^2^ = 8.402, *p* = 0.004). This finding is in line with previous evidence showing that long-stay patients are often associated with poorer health and more likely to receive invasive procedures or specific treatments, which may contribute to an increase in HAP (34). It suggests that the middle-aged and elderly male patients with schizophrenia who have been hospitalized for ≥60 days should be regarded as the key population for active HAP prevention.

### Antipsychotic Drug Influencing Factors of HAP

The sedation and muscle relaxation effects of antipsychotics inhibit the movement function of respiratory cilia and weakened the ability of the respiratory tract to clear pathogenic bacteria, resulting in increases in pneumonia risk and HAP incidence (35). However, the effect of antipsychotic drug type on pneumonia remains controversial. Some studies (36) have reported that the use of FGA increases the incidence of acute pneumonia in hospitalized patients and leads to an increased risk of death. Others have found that SGA is associated with HAP and clozapine is particularly associated with a higher risk of pneumonia (37). There is also evidence suggesting that the use of either FGA or SGA in elderly patients with mental illness leads to an increased risk of pneumonia (35, 38).

Our study showed that SGA (quetiapine, clozapine, and olanzapine), rather than FGA, was associated with an increased risk of HAP after excluding the interaction between drugs using multivariate conditional logistic regression.

Previous research (37) has suggested that the association between pneumonia and antipsychotics may be mediated by the affinity of the drugs to the muscarinic 1 (M1) and histaminergic 1 (H1) receptor. Antipsychotic drugs competitively bind to the M1 receptor and block the M1 receptor from binding to acetylcholine, bringing about anticholinergic effects. Then, dry mouth, esophagus dilation, reduced paraperistalsis, and reflux of gastric contents eventually lead to aspiration pneumonia (39). Anticholinergic action can also cause bronchial mucus to thicken in the respiratory system, which aggravates bronchitis. Studies (35, 37, 38) have shown that clozapine has the highest affinity with M1 receptor while olanzapine and quetiapine have moderate affinity. In the present study, clozapine had the highest risk ratio for pneumonia (OR = 1.81), followed by olanzapine (OR = 1.68) and quetiapine (OR = 1.56) after adjustment for other interference factors. This also confirms that the affinity of drugs to M1 receptor does have a certain correlation with HAP. Similarly, antipsychotics that antagonize H1 receptors in the central nervous system and lead to oversedation and salivation may also contribute to the development of aspiration pneumonia (40). Drugs (quetiapine, clozapine, and olanzapine) that were strongly associated with HAP (OR > 1.5) here happen to have a high affinity for the H1 receptor, which verifies the previous conclusions about the association between HAP and the H1 receptor (37).

Plenty of studies have shown that clozapine may cause more cases of pneumonia and higher mortality than other antipsychotics (41). The possible mechanisms are as follows: (1) Clozapine is more likely to cause oversedation and salivation than olanzapine and quetiapine, thus leading to a higher risk of aspiration pneumonia (42); (2) Clozapine-induced agranulocytosis/granulocytopenia (CIAG) (43), may be contributed by the immune-mediated response against haptenized neutrophils. Within the therapeutic doses range of clozapine, the proliferation of peripheral blood mononuclear cells is stimulated (44). Another potential mechanism is direct toxicity against bone marrowstromal cells (45), as well as immaturity of the neutrophil population (46); (3) Other adverse reactions (ADRs), such as intestinal obstruction caused by severe constipation and myocarditis, may be complicated with pneumonia (47). In addition, some studies have shown a strong bi-directional association between clozapine and pneumonia. When severe inflammation occurs, the metabolism of clozapine is reduced, which enhances serum clozapine concentration and further increases the risk of serum concentration related ADRs, including excessive sedation, salivation, aspiration, and even arrhythmias, resulting in a very dangerous positive feedback (47). de Leon (48) has recommended halving clozapine doses during periods of severe infection, including pneumonia, until a normal clozapine concentration is achieved.

Either olanzapine or quetiapine is an SGA similar to clozapine (49). A systemic review (50) showed that olanzapine, a new generation of antipsychotic drugs derived from clozapine, significantly reduced the side effects of oversedation, seizures, and granulocytopenia. However, olanzapine did not differ from clozapine in cardiac effects, mortality, extrapyramidal reactions (EPS), and weight gain (51). Therefore, olanzapine is also associated with a higher risk of HAP.

Although quetiapine is ideal for the treatment of schizophrenia and has fewer effects on patients' blood lipid, glucose metabolism, and body weight than clozapine or olanzapine (52), there are still side effects such as drowsiness, postural hypotension, palpitations, and dry mouth, which lead to an increased risk of HAP.

In this study, FGA use was not significantly associated with HAP, but the haloperidol group had a lower incidence of HAP. Haloperidol is the main representative of the FGA of butylbenzene. At the same dose, its antagonistic effect on dopamine receptors is 20~40 times that of chlorpromazine and therefore it is a strong and low-dose antipsychotic drug. It is usually used shortly at the beginning of an acute episode of mental illness and discontinued after rapid control of symptoms. Thus, the most prominent EPS to FGA can be reduced or eliminated with short doses of the drug, reducing the risk of HAP.

### Non-antipsychotic Risk Factors for HAP

Our results showed that non-antipsychotic drugs, such as aceglutamide, were associated with HAP, and this association persisted after controlling for other factors. Aceglutamide is used for adjuvant treatment of senile brain function decline. It passes the blood-cerebrospinal fluid barrier and breaks down into glutamate and γ-aminobutyric acid (GABA). GABA binds to GABA receptors and inhibits post-synaptic neuronal excitation. Aceglutamide injection was used in this study. When the infusion is too fast or too large, the blood concentration of the drug increases rapidly, which stimulates the norepinephrine neurons located in the ventral lateral part of the medulla oblongata, thereby inhibiting the neuronal activity of cardiac sympathetic constrictive nerve and leading to vasodilation, drop of blood pressure, and even hypovolemic shock. This may contribute to aspiration pneumonia in patients. Therefore, physicians should carefully choose adjuvant drugs apart from those for the control of patients' specialized diseases in clinical use, so as to prevent new adverse effects on patients.

## Conclusion

In the retrospective study, we observed 2,617 inpatients aged 50 and older with schizophrenia in a large-scale psychiatric hospital from 2016 to 2020, and drew the following conclusions. Male patients with schizophrenia who are over 50 years old and have been hospitalized for more than 60 days may be at high risk for HAP in psychiatric institutions. While using SGA (quetiapine, clozapine, and olanzapine) drugs with caution, clinicians should also focus on monitoring patients for pulmonary infection. In addition, the use of adjuvant drugs such as aceglutamide should be minimized. However, this conclusion is limited by the lack of data on subjects including comorbidities, combination and dosage of antipsychotic drugs, and symptoms of schizophrenia in the literature.

## Limitations

First of all, this study involves only one mental institution and data from multiple centers of different sizes should be included in the future for a hierarchical analysis. Secondly, there was a lack of interesting data on comorbidities, symptoms, and combination and dosage of antipsychotic drugs in patients with schizophrenia. The collection of the data may help understand the influence of antipsychotics on HAP from all aspects. Finally, this is a retrospective study and a prospective study may need to be designed to validate our results.

## Data Availability Statement

The raw data supporting the conclusions of this article will be made available by the authors, without undue reservation.

## Ethics Statement

The studies involving human participants were reviewed and approved by Institutional Review Committee of the Fourth People's Hospital of Chengdu. Written informed consent for participation was not required for this study in accordance with the national legislation and the institutional requirements.

## Author Contributions

MY and QL searched and reviewed the literature, analyzed the data, and wrote the manuscript. CW, LL, and MX collected and analyzed the data. FY, WC, and YW searched the literature and collected data. All authors contributed to the article and approved the submitted version.

## Funding

This study was supported by the National Natural Science Foundation of China (grant numbers 61806042 and 62073058); the Special Research Project for the novel coronavirus pneumonia funded by the Chengdu Science and Technology Bureau (grant number 2020-YF05-00171-SN); and the Science and Technology Department of Sichuan Province (grant number 2018sz0236).

## Conflict of Interest

The authors declare that the research was conducted in the absence of any commercial or financial relationships that could be construed as a potential conflict of interest.

## Publisher's Note

All claims expressed in this article are solely those of the authors and do not necessarily represent those of their affiliated organizations, or those of the publisher, the editors and the reviewers. Any product that may be evaluated in this article, or claim that may be made by its manufacturer, is not guaranteed or endorsed by the publisher.
